# Clinical features of chronic hepatitis B patients with low hepatitis B surface antigen levels and determinants of hepatitis B surface antigen seroclearance

**DOI:** 10.1002/jgh3.12321

**Published:** 2020-03-12

**Authors:** Hideaki Taniguchi, Yoshiaki Iwasaki, Masahito Aimi, Gaku Shimazaki, Akio Moriya

**Affiliations:** ^1^ Internal Medicine Tottori Municipal Hospital Tottori Japan; ^2^ Health Service Center Okayama University Okayama Japan; ^3^ Gastroenterology Mitoyo General Hospital Japan

**Keywords:** aging, hepatitis B surface antigen, hepatitis B surface antigen seroclearance, hepatitis B virus DNA

## Abstract

**Background and Aim:**

A low hepatitis B surface antigen (HBsAg) level is reported to be predictive of future HBsAg seroclearance. A hospital‐based cohort study was conducted to clarify the clinical features of patients with low HBsAg levels and to demonstrate the usefulness of low HBsAg levels for predicting HBsAg seroclearance.

**Methods:**

A total of 1459 patients with chronic hepatitis B were included in the study. Of these, 587 had repeated measurements for HBsAg levels and two or more records of HBsAg‐positive results. HBsAg levels were measured with a commercially available HBsAg assay. Based on a cut‐off index (COI) of 2000, a high HBsAg level was defined as HBsAg ≥2000 COI, and a low HBsAg level was defined as HBsAg <2000 COI.

**Results:**

The proportion of patients with low HBsAg levels at baseline tended to increase with age. Patients with low HBsAg levels at baseline had significantly older age, lower transaminase levels, and lower hepatitis B virus (HBV) DNA levels than those with high HBsAg levels. The annual HBsAg seroclearance rate was 1.30%/year. The cumulative incidences of HBsAg seroclearance differed significantly by HBsAg level at baseline (<2000 *vs* ≥2000 COI), age (≥50 *vs* <50 years), and HBV DNA level (<4.0 *vs* ≥4.0 log copies/mL). Cox proportional hazards regression analyses showed that low HBsAg level (<2000 COI) and low HBV DNA level (<4.0 log copies/mL) were significantly associated with HBsAg seroclearance.

**Conclusion:**

Aging was one of the factors affecting HBsAg level. HBsAg seroclearance was significantly associated with low HBsAg level and low HBV DNA level at baseline.

## Graphical abstract

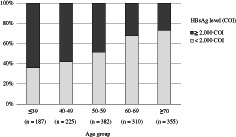

The proportion of high HBsAg level tended to decrease with age. Meanwhile, the proportion of low HBsAg level tended to increase with age. Increasing age is a major determinant of HBsAg level.

## Introduction

Hepatitis B virus (HBV) infection is the most common chronic viral infection worldwide and represents a major public health problem.^1^ It is estimated that, in 2015, 257 million people were living with chronic hepatitis B (CHB) infection.^2^

In the management of CHB, quantification of hepatitis B surface antigen (HBsAg) level is known to provide valuable information in certain clinical situations. For example, the risk of developing hepatocellular carcinoma (HCC) increases with increasing HBsAg level among hepatitis B e antigen (HBeAg)‐negative patients with low HBV DNA levels,^3^, ^4^ and a low HBsAg level (<1000 IU/mL) in combination with a low HBV DNA level (<2000 IU/mL) is effective for distinguishing inactive carriers from active carriers with high accuracy.^5^, ^6^ Furthermore, low HBsAg level was identified as a predictive factor for HBsAg seroclearance,^7^, ^8^, ^9^, ^10^, ^11^ which is regarded as a functional cure of CHB^12^ and a goal of CHB treatment.^13^, ^14^, ^15^ Considered together, CHB patients with low HBsAg levels are likely to have a favorable clinical course, and low HBsAg level may be an optimistic indicator. However, conflicting data have also been reported. A hospital‐based study in Japan demonstrated that HCC risk was significantly higher in patients with low HBsAg level (<1000 IU/mL) than in patients with high HBsAg level (≥1000 IU/mL).^16^ In addition, cirrhotic patients were reported to have significantly lower HBsAg levels than noncirrhotic patients.^17^ Furthermore, HBsAg level tended to decrease with liver fibrosis stage in HBeAg‐positive patients,^18^, ^19^, ^20^ and no clear association between HBsAg level and biopsy‐proven liver fibrosis stage was found in HBeAg‐negative patients.^21^ To the best of our knowledge, there are few studies on factors that affect HBsAg levels.

In this study, we carried out a hospital‐based study to examine the clinical features of CHB patients with low HBsAg levels and to clarify the predictive factors for HBsAg seroclearance in an HBV genotype C‐dominant region in Japan.

## Methods

### *Study cohort*

**Figure 1:**
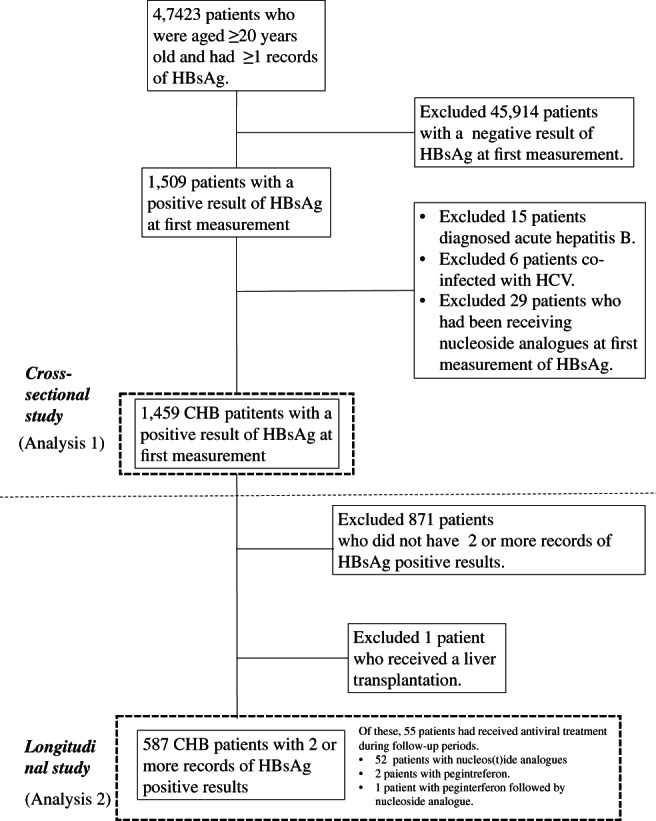
Flow of patients included in the study. A cross‐sectional study (analysis 1) was performed using data for 1459 CHB patients at first measurement of HBsAg level. A longitudinal study (analysis 2) was performed using data for 587 CHB patients with two or more records of HBsAg‐positive results. Of 587 patients, 55 received antiviral therapy with interferon or nucleos(t)ide analogues during the follow‐up periods. CHB, chronic hepatitis B; HBsAg, hepatitis B surface antigen; HCV, hepatitis C virus.

From April 2003 to September 2016, 47 423 consecutive patients aged ≥20 years who underwent HBsAg measurements at least once at Tottori Municipal Hospital were recruited. Of these, 1509 patients (3%) were seropositive for HBsAg at first measurement. The following patients were excluded from the study: 15 patients diagnosed with acute hepatitis B, 6 patients coinfected with hepatitis C virus (HCV), and 29 patients who were receiving nucleoside analogues at first HBsAg measurement. Finally, 1459 patients with CHB were included in the study. Of these, 587 had repeated measurements of HBsAg levels and two or more records of HBsAg‐positive results (Fig. 1). Of the 587 patients, 55 had received antiviral therapy during the follow‐up periods: interferon (*n* = 2), nucleot(s)ide analogues (*n* = 52), and interferon followed by nucleoside analogue (*n* = 1) (Fig. 1).

We retrospectively analyzed the data of the included patients. The study conformed to the ethical guidelines in the Declaration of Helsinki (2000 version) and was approved by the Ethics Committee at the institution.

### *Data collection*

Baseline data were defined as data at first measurement of HBsAg. Demographic characteristics including gender and birth data were collected. Baseline data for liver biochemistry tests and hematological and virological parameters were also collected. Thereafter, for patients with repeated HBsAg measurements, serial data for HBsAg levels, as well as liver biochemistry tests and hematological and virological parameters, were collected until last follow‐up of HBsAg.

### *Measurement of HBsAg levels*

HBsAg levels were measured by the Lumipulse II HBsAg assay (Fujirebio, Tokyo, Japan). In the manufacturer's guidelines, the cut‐off value was ≥1.0 cut‐off index (COI), and the upper measurable limit was 2000 COI. Although this assay is semiquantitative, previous studies have demonstrated that the Lumipulse II HBsAg is a highly sensitive and commercially available quantitative assay.^13^, ^22^ Therefore, high HBsAg level was defined as HBsAg ≥2000 COI, and low HBsAg level was defined as HBsAg <2000 COI, as previously reported.^23^

### *Measurement of HBV DNA levels*

HBV DNA levels were measured by transcription‐mediated amplification (TMA) (from April 2003 to April 2015) or polymerase chain reaction (PCR) (from April 2015 to September 2016). Because of the excellent correlation between the two methods, HBV DNA 1 log genome equivalent/mL by TMA was considered equivalent to HBV DNA 1 log copy/mL by PCR.

### *Definition of HBsAg seroclearance and age at HBsAg seroclearance*

During the follow‐up period, patients with a negative HBsAg result (<1.0 COI) following a positive HBsAg result at baseline were determined to have achieved HBsAg seroclearance. Age at HBsAg seroclearance was estimated as mean age between last HBsAg‐detectable age and first HBsAg‐undetectable age.

### *Estimation of presence of cirrhosis*

Instead of a liver biopsy, the presence of cirrhosis was estimated by three fibrosis indices: platelet count,^24^ aspartate aminotransferase‐to‐platelet ratio index (APRI),^25^ and fibrosis index based on four factors (Fib4 index).^26^ The cut‐off values for predicting cirrhosis were based on previous findings: platelet count ≤10 × 10^4^/mm^3^,^24^ APRI >2.0,^25^, ^27^, ^28^ and Fib4 index >3.6.^28^, ^29^

### *Statistical analysis*

Continuous variables were expressed as median (range), and discrete variables were expressed as frequency (percentage). Qualitative and quantitative differences between two groups were analyzed by the chi‐square test for categorical parameters and the Mann–Whitney test for continuous parameters, as appropriate. Qualitative and quantitative differences between three or more subgroups were analyzed by the chi‐square test for categorical parameters and the Kruskal–Wallis test for continuous parameters, as appropriate.

To examine the association between age and HBsAg level, we categorized age and HBsAg into groups as follows. Age at first measurement of HBsAg level was categorized into five groups: ≤39 (*n* = 187), 40–49 (*n* = 225), 50–59 (*n* = 382), 60–69 (*n* = 310), and ≥70 (*n* = 355) years. The increasing or decreasing trends in HBsAg levels were evaluated by the Cochran–Armitage trend test for categorical data. The cumulative incidence of HBsAg seroclearance was assessed by the Kaplan–Meier method and compared between variables at baseline by the log‐rank test. Cox proportional hazards models were used to analyze the crude and multivariate‐adjusted hazard ratios (HRs) and 95% confidence intervals (CIs) of each factor for HBsAg seroclearance. All tests were two‐sided and had a significance level of 0.05. Data handling and analysis were performed with EZR software version 1.40 for Windows.^30^

## Results

### *Cross‐sectional study (analysis 1)*

#### *Characteristics of the study patients*

Table 1 shows the characteristics of the 1459 patients at first measurement of HBsAg level. Of the patients, 835 were male (57.2%). Median age was 58 years (range: 20–99 years). High HBsAg level (≥2000 COI) was observed in 633 patients (43.4%). Median alanine aminotransferase (ALT) level was 22 IU/L, and most patients had ALT levels below 30 IU/L (69.2%). Median values of platelet count, APRI, and Fib4 index were 20.4 × 10^4^/mm^3^, 0.30, and 1.52, respectively. Although not measured in all patients, most patients had post‐HBeAg seroconversion status (90.2%) and HBV DNA levels below 4.0 log copies/mL (61.6%).

**Table 1:** Patient characteristics at baseline and comparison between patients in the high HBsAg group and low HBsAg group

Variable	Total *n* †		Comparison based on HBsAg levels		
			Low HBsAg group (HBsAg <2000 COI)	High HBsAg group (HBsAg ≥2000 COI)	*P*‐value
Male, *n* (%)	1459	835 (57.2)	489 (59.2)	346 (54.7)	0.088
Female, *n* (%)		624 (42.8)	337 (40.8)	287 (45.3)	
Age, years, median (range)	1459	58 (20–99)	63 (22–99)	53 (20–95)	<0.001
Age, years	1459				
≤39, *n* (%)		187 (12.8)	67 (8.1)	120 (19.0)	<0.001
40–49, *n* (%)		225 (15.4)	94 (11.4)	131 (20.7)	
50–59, *n* (%)		382 (26.2)	197 (23.8)	185 (29.2)	
60–69, *n* (%)		310 (21.2)	209 (25.3)	101 (16.0)	
≥70, *n* (%)		355 (24.3)	259 (31.4)	96 (15.2)	
HBsAg level	1459				
≥2000 COI		633 (43.4)			
<2000 COI		826 (56.6)			
AST, IU/L, median (range)	1435	24 (7–3194)	24 (7–2560)	25 (10–3194)	<0.001
AST	1435				
<30 IU/L, *n* (%)		998 (69.5)	587 (72.3)	411 (66.0)	0.011
≥30 IU/L, *n* (%)		437 (30.5)	225 (27.7)	212 (34.0)	
ALT, IU/L, median (range)	1435	22 (6–2830)	20 (6–746)	24 (6–2830)	<0.001
ALT	1435				
<30 IU/L, *n* (%)		993 (69.2)	598 (73.6)	395 (63.4)	<0.001
≥30 IU/L, *n* (%)		442 (30.8)	214 (26.4)	228 (36.6)	
Platelet count, median (range)	1443	20.4 (1.7–60.0)	20.7 (2.9–60.0)	19.8 (1.7–43.6)	0.106
Platelet count	1443				
≤10 × 10^4^/mm^3^, *n* (%)		67 (4.6)	34 (4.1)	33 (5.3)	0.313
>10 × 10^4^/mm^3^, *n* (%)		1376 (95.4)	790 (95.9)	586 (94.7)	
Platelet count	1443				
≤15 × 10^4^/mm^3^, *n* (%)	1443	237 (16.4)	129 (15.7)	108 (17.4)	0.389
>15 × 10^4^/mm^3^, *n* (%)		1206 (83.6)	695 (84.3)	511 (82.6)	
HBV DNA	380				
<4.0 log copies/mL, *n* (%)	380	234 (61.6)	141 (83.4)	93 (44.1)	<0.001
≥4.0 log copies/mL, *n* (%)		146 (38.4)	28 (16.6)	118 (55.9)	
HBeAg	488				
+, *n* (%)		48 (9.8)	0 (0.0)	48 (17.0)	<0.001
−, *n* (%)		440 (90.2)	206 (100.0)	234 (83.0)	
HBeAb	485				
+, *n* (%)		430 (88.7)	191 (93.2)	239 (85.4)	0.009
−, *n* (%)		55 (11.3)	14 (6.8)	41 (14.6)	
APRI	1426				
≤2.0, *n* (%)		1366 (95.8)	781 (96.2)	585 (95.3)	0.426
>2.0, *n* (%)		60 (4.2)	31 (3.8)	29 (4.7)	
Fib4 index	1426				
≤3.6, *n* (%)		1282 (89.9)	729 (89.8)	553 (90.1)	0.929
>3.6, *n* (%)		144 (10.1)	83 (10.2)	61 (9.9)	

† Number of patients with available data at first measurement of HBsAg level (at baseline).

ALT, alanine aminotransferase; APRI, aspartate aminotransferase‐to‐platelet ratio index; AST, aspartate aminotransferase; COI, cut‐off index; Fib4 index, fibrosis index based on four factors; HBeAb, hepatitis B e antibody; HBeAg, hepatitis B e antigen; HBsAg, hepatitis B surface antigen; HBV, hepatitis B virus.

#### *Inverse correlation between HBsAg level and increasing age*

**Figure 2:**
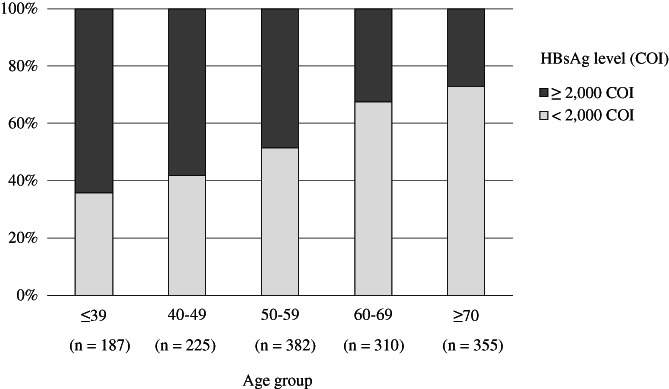
Association between HBsAg level and age. The proportion of patients with HBsAg level ≥2000 COI tended to decrease with age (64.2, 58.2, 48.4, 32.6, and 27.0% for age groups ≤39, 40–49, 50–59, 60–69, and ≥70 years, respectively; *P* < 0.001, Cochran–Armitage test for trend). 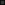, HBsAg level ≥2,000 COI; 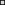, HBsAg level <2,000 COI. COI, cut‐off index; HBsAg, hepatitis B surface antigen.

We analyzed the correlation between age and HBsAg level. HBsAg level was divided into two groups: HBsAg ≥2000 COI (*n* = 633) and HBsAg <2000 COI (*n* = 826). The proportion of patients with HBsAg ≥2000 COI tended to decrease with age (64.2, 58.2, 48.4, 32.6, and 27.0% for age groups ≤39, 40–49, 50–59, 60–69, and ≥70 years, respectively; *P* < 0.001, Cochran–Armitage test for trend; Fig. 2).

#### *Comparison between patients with high and low HBsAg levels*

We compared the demographic characteristics and laboratory data between patients with high (≥2000 COI) and low (<2000 COI) HBsAg levels (Table 1). Patients with low HBsAg levels were significantly older than those with high HBsAg levels (63 years *vs* 53 years, *P* < 0.001). Median aspartate aminotransferase (AST) and ALT levels were significantly lower in patients with low HBsAg levels than in those with high HBsAg levels (AST: 24 IU/L *vs* 25 IU/L, *P* < 0.001; ALT: 20 IU/L *vs* 24 IU/L, *P* < 0.001). The prevalence rates of platelet count ≤15 × 10^4^/mm^3^ or ≤10 × 10^4^/mm^3^, indicating advanced fibrosis and cirrhosis, respectively, did not differ between the two groups. Similarly, the ratios of estimated cirrhosis defined as APRI >2.0 or Fib4 index >3.6 did not differ between the two groups. Regarding HBeAg and hepatitis B e antibody (HBeAb) status, patients with high HBsAg levels had a higher ratio of HBeAg‐positive results than those with low HBsAg levels (17.0 *vs* 0%, *P* < 0.001). Meanwhile, patients with low HBsAg levels had a higher ratio of HBeAb‐positive results than those with low HBsAg levels (93.2 *vs* 85.4%, *P* = 0.009). The prevalence of patients with HBV DNA <4.0 log copies/mL was significantly higher in patients with low HBsAg levels than in those with high HBsAg levels (83.4 *vs* 44.1%, *P* < 0.001).

### *Longitudinal study (analysis 2)*

#### *Longitudinal follow‐up of HBsAg levels: Annual HBsAg seroclearance rate in person‐years analysis and predictive factors associated with future HBsAg seroclearance*

**Figure 3:**
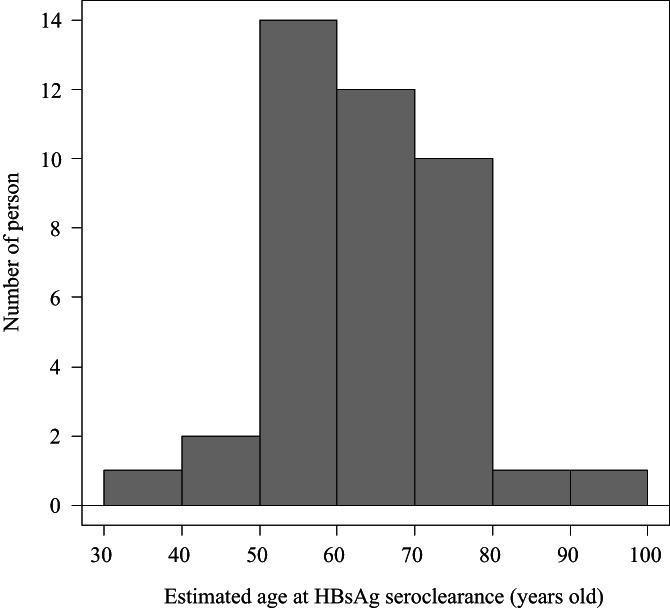
Frequency of HBsAg seroclearance and estimated age at seroclearance. In this cohort, 41 patients achieved HBsAg seroclearance during 3163.8 person‐years of follow‐up. Median estimated age at HBsAg seroclearance was 63 years, and 87% of patients with HBsAg seroclearance were between 50 and 80 years of age. HBsAg, hepatitis B surface antigen.

The 587 patients had 3163.8 person‐years of follow‐up. During the follow‐up period, 41 achieved HBsAg seroclearance. The annual HBsAg seroclearance rate was estimated to be 1.30%/year. Median estimated age at HBsAg seroclearance was 63 years, and 87% of patients with HBsAg seroclearance were between 50 and 80 years of age (Fig. 3).

**Figure 4:**
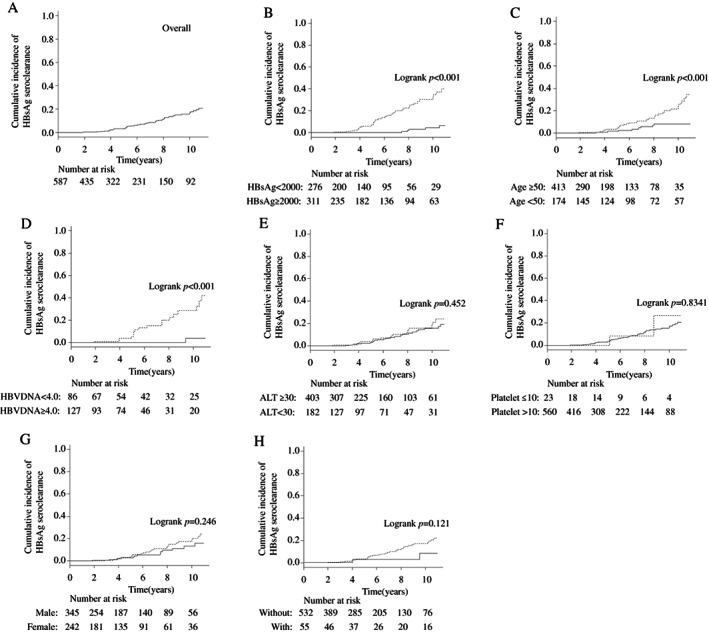
Cumulative incidences of HBsAg seroclearance in 587 patients in the cohort. (a) The cumulative incidences of HBsAg seroclearance were 3.2 and 16.6% after 5 and 10 years, respectively. (b–h) Cumulative incidences of HBsAg seroclearance according to the following variables at baseline: (b) HBsAg level (<2000 *vs* ≥2000 COI)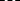, HBsAg <2,000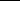, HBsAg ≥2,000; (c) age (<50 *vs* ≥50 years)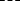, age ≥50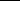, age <50; (d) HBV DNA level (<4.0 *vs* ≥4.0 log copies/mL)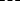, HBVDNA <4.0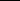, HBVDNA ≥4.0; (e) ALT (≥30 *vs* <30 IU/L)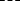, ALT ≥30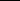, ALT<30; (f) platelet count (≤10 *vs* >10 × 10^4^/mm^3^)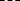, platelet ≤10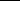, platelet >10; (g) gender (male *vs* female)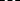, male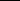, female; (h) antiviral therapy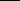, without antiviral therapy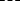, with antiviral therapy. The cumulative incidence of HBsAg seroclearance was significantly higher in patients with low HBsAg level (<2000 COI), high age (≥50 years), and low HBV DNA level (<4.0 log copies/mL) (log‐rank *P* < 0.001, *P* < 0.001, and *P* < 0.001, respectively). Meanwhile, the cumulative incidences of HBsAg seroclearance did not differ by ALT level, platelet count, gender, and antiviral therapy. ALT, alanine aminotransferase; COI, cut‐off index; HBsAg, hepatitis B surface antigen; HBV, hepatitis B virus.

As shown in Figure 4a, the cumulative incidences of HBsAg seroclearance were 3.2 and 16.6% after 5 and 10 years, respectively. The cumulative incidences of HBsAg seroclearance by HBsAg level, age, gender, platelet count, ALT, HBV DNA level, and antiviral therapy during follow‐up periods are also shown in Figure 4. The cumulative incidence of HBsAg seroclearance was significantly higher in patients with low HBsAg level (<2000 COI), high age (≥50 years), and low HBV DNA level (<4.0 log copies/mL) (log‐rank *P* < 0.001, *P* < 0.001, and *P* < 0.001, respectively; Fig. 4b–d). The cumulative incidences of HBsAg seroclearance did not differ by ALT level, platelet count, gender, and antiviral therapy during follow‐up periods (Fig. 4e–h).

Cox proportional hazards regression analyses including two significant factors, age (≥50 years) and low HBsAg level (<2000 COI), showed that low HBsAg level (<2000 COI), but not age (≥50 years), was significant for HBsAg seroclearance (Table 2). Cox proportional hazards regression analyses including three significant factors, age (≥50 years), low HBsAg level (<2000 COI), and low HBV DNA level (<4.0 log copies/mL), showed that low HBsAg level (<2000 COI) and low HBV DNA (<4.0 log copies/mL) were significant for HBsAg seroclearance, although the analysis was incomplete due to a substantial number of patients with missing data for HBV DNA levels (Table 2).

**Table 2:** Cox proportional hazards regression analyses for factors predictive of HBsAg seroclearance

Factors	Univariate analysis		Multivariate analysis (model 1)†		Multivariate analysis (model 2)‡		Multivariate analysis (model 3)§	
	Hazard ratio (95% CI)	*P*‐value	Hazard ratio (95% CI)	*P*‐value	Hazard ratio (95% CI)	*P*‐value	Hazard ratio (95% CI)	*P*‐value
Age ≥50 years old	3.57(1.63–7.8)	0.001	2.06 (0.92–4.57)	0.077			3.43 (0.76–15.41)	0.108
Low HBsAg level (<2000 COI)	11.17 (4.38–28.51)	<0.001	9.32 (3.59–24.19)	<0.001	6.86 (1.97–23.97)	0.003	5.25 (1.47–18.72)	0.011
Low HBV DNA (<4.0 log copies/mL)¶	18.12 (2.41–136.2)	0.005			11.19 (1.46–85.74)	0.020	9.25 (1.20–71.34)	0.033

† Multivariate analysis (Model 1) including two factors of age and HBsAg level.

‡ Multivariate analysis (Model 2) including two factors of HBsAg level and HBV DNA level, although this analysis was performed with available data for HBV DNA level.

§ Multivariate analysis (Model 3) including three factors of age, HBsAg level, and HBV DNA level, although this analysis was performed with available data for HBV DNA level.

¶ Including 374 patients with available data for HBV DNA level.

CI, confidence interval; COI, cut‐off index; HBsAg, hepatitis B surface antigen; HBV, hepatitis B virus.

## Discussion

This study clearly showed a significant inverse correlation between HBsAg level and age, indicating that increasing age is a major determinant of HBsAg levels. Thus, higher age was associated with lower HBsAg level. Although the precise mechanism for why increasing age is correlated with decreasing HBsAg level remains unclear, a possible explanation could be immune elimination of HBV‐infected hepatocytes. Most patients with chronic HBV infection in Japan are infected with HBV through vertical transmission at delivery or at age below 6 years, and thus, HBV‐infected hepatocytes containing HBV covalently closed circular DNA may be decreased through a long‐term immune response. Another explanation may be HBV preS/S gene mutations leading to reduced HBsAg secretion. Indeed, Pollicino and colleagues reported that patients infected with preS/S mutant HBV had lower HBsAg levels than patients infected with wild‐type HBV.^31^, ^32^ Further studies are required to evaluate the presence of preS/S mutant HBV or ratio of preS/S mutant HBV to wild‐type HBV in association with HBsAg level.

Low HBsAg level was identified as a clinical factor that can predict favorable outcomes in terms of interferon therapeutic effects^33^, ^34^, ^35^ and HBsAg seroclearance. Therefore, the present study compared the clinical features between patients with high (≥2000 COI) and low (<2000 COI) HBsAg levels (Table 1). We found that patients with low HBsAg levels had significantly older age, lower transaminase levels, and lower HBV DNA levels than those with high HBsAg levels. These findings suggest that a low HBsAg level is likely to be associated with inactive HBV carrier state and occurs with aging. However, platelet count, Fib4 index, and APRI, with cut‐off values set for the prediction of cirrhosis, did not differ between the two groups. These results indicate that patients with low HBsAg levels do not necessarily have mild hepatic fibrosis. Liver fibrosis progression in CHB may be the consequence of multiple complex factors, including severity and duration of active hepatitis phase, aging, hepatic steatosis, diabetes, and alcohol drinking, among others.^36^, ^37^, ^38^ Given that patients with low HBsAg levels had an obviously higher age, various factors may contribute to liver fibrosis progression over time along with decreases in HBsAg level.

In accordance with previous studies,^7^, ^39^, ^40^, ^41^ we found that age ≥50 years at baseline, low HBsAg level, and low HBV DNA level were significantly associated with HBsAg seroclearance in univariate analyses. Multivariate analyses demonstrated that HBsAg level and HBV DNA level, but not age, were independently associated with HBsAg seroclearance. A possible explanation for these findings is that HBsAg level was significantly correlated with age, as shown in Figure 2, indicating that HBsAg level was more important for the prediction of HBsAg seroclearance than age. Furthermore, previous studies demonstrated a positive correlation between HBsAg level and HBV DNA level, but the correlation became dissociated as the clinical phase progressed from immune tolerance to inactive carrier.^17^, ^42^ Because the majority of patients who achieved HBsAg seroclearance were inactive carriers, both low HBsAg level and low HBV DNA level could be independent significant factors for HBsAg seroclearance.

We did not find any associations of platelet count, APRI, and Fib4 index at baseline (data not shown for APRI and Fib4 index) with HBsAg seroclearance. These findings suggest that liver fibrosis stage may not have a definitive effect on HBsAg seroclearance.

In this study, HBsAg seroclearance occurred at a relatively high age compared with previous studies. Specifically, the median age at HBsAg seroclearance was 63 years in the present study compared with 48 years in a study from Taiwan^43^ and 49.6 years in a study from Hong Kong.^44^ Although the reason for the difference remains unclear, it may be partly attributable to the difference in HBV genotypes. Japanese HBV carriers are known to be predominantly infected with genotype C, while Taiwanese HBV carriers are predominantly infected with genotype B. HBV genotype B was reported to be more strongly associated with HBsAg seroclearance than HBV genotype C.^43^, ^45^ Other reasons may be the difference in age distributions—because our cohort included a large number of elderly patients—or the nature of a hospital‐based study. It is worth noting that HBsAg seroclearance was observed to occur in elderly patients aged ≥50 years (Fig. 3). Nevertheless, several previous studies demonstrated that benefits from HBsAg seroclearance were fewer in patients aged ≥50 years at the time of HBsAg seroclearance.^44^, ^46^, ^47^, ^48^, ^49^ Therefore, maintenance of surveillance for HCC emergence may be required despite the achievement of HBsAg seroclearance,

It is not easy to distinguish between CHB patients after HBsAg seroclearance and with prior transient HBV infection because both are negative for HBsAg and positive for hepatitis B core antibody and/or hepatitis B surface antibody. However, HBV DNA is known to be more frequently detectable in the serum and liver tissue of former patients.^44^, ^50^ Considering the lasting detectability of HBV DNA, HBV reactivation in the setting of cancer chemotherapy and immunosuppressive therapy is more likely to occur in CHB patients after HBsAg seroclearance. Tanaka *et al*.^51^ reported that approximately 900 000 individuals were estimated to have undiagnosed HBV infection in Japan in 2005. A recent meta‐analysis demonstrated that the annual HBsAg seroclearance rate was 1.02% and increased up to 17.99% after 15 years.^52^ Based on these findings, there may be a considerable number of CHB patients after HBsAg seroclearance who are unaware of the past presence of HBsAg‐positive CHB. Therefore, it should be emphasized that careful attention is needed to prevent HBV reactivation in the setting of cancer chemotherapy and immunosuppressive therapy, especially in HBV epidemic areas.

There are some limitations to the present study. First, because liver biopsy was not a routine procedure, we had no histological proof of the hepatic fibrosis stage. Second, HBsAg levels were monitored using a semiquantitative assay and thus could not be represented as international units (IU/mL). Third, because this study had a retrospective nature, the significant amount of missing laboratory data may have weakened the statistical power in the multivariate analyses.

In summary, we demonstrated that HBsAg level was inversely correlated with age, indicating a gradual decreasing trend in HBsAg level with increasing age. The association of low HBsAg level with higher age and lower aminotransferase levels suggests that low HBsAg level may be associated with inactive HBV carrier state and may occur with aging. In this cohort study, the annual HBsAg seroclearance rate was 1.30%/year, and both low HBsAg level and low HBV DNA level at baseline were predictive factors for HBsAg seroclearance.
